# Cerebrospinal Fluid Spermidine, Glutamine and Putrescine Predict Postoperative Delirium Following Elective Orthopaedic Surgery

**DOI:** 10.1038/s41598-019-40544-3

**Published:** 2019-03-12

**Authors:** Xiaobei Pan, Emma L. Cunningham, Anthony P. Passmore, Bernadette McGuinness, Daniel F. McAuley, David Beverland, Seamus O’Brien, Tim Mawhinney, Jonathan M. Schott, Henrik Zetterberg, Brian D. Green

**Affiliations:** 1 Institute for Global Food Security, School of Biological Sciences, Queen’s University Belfast, 8 Malone Road, Belfast, BT9 5BN Northern Ireland; 2 Centre for Public Health, Queen’s University Belfast, Block B, Institute of Clinical Sciences, Royal Victoria Hospital site, Grosvenor Road, Belfast, BT12 6BA Northern Ireland; 3 Centre for Experimental Medicine, Queen’s University Belfast, Wellcome-Wolfson Institute for Experimental Medicine, 97 Lisburn Road, Belfast, BT9 7BL Northern Ireland; 4 Outcomes Assessment Unit, Musgrave Park Hospital, Belfast Trust, Stockman’s Lane, Belfast, BT9 7JB Northern Ireland; 5 Cardiac Surgical Intensive Care Unit, Belfast Trust, Royal Victoria Hospital, Grosvenor Road, Belfast, BT12 6BA Northern Ireland; 6 Dementia Research Centre, Department of Neurodegenerative Disease, UCL Institute of Neurology, UK, Box 16, National Hospital for Neurology and Neurosurgery, Queen Square, London, WC1N 3BG UK; 7 UK Dementia Research Institute at UCL, Cruciform Building, Gower Street, London, London, WC1E 6BT UK; 8 Department of Neurodegenerative Disease, UCL Institute of Neurology, London, UK, Box 16, National Hospital for Neurology and Neurosurgery, Queen Square, London, WC1N 3BG UK; 9 Clinical Neurochemistry Laboratory, Sahlgrenska University Hospital, House V, S-431 80 Mölndal, Göteborg, Sweden; 10 Department of Psychiatry and Neurochemistry, Institute of Neuroscience and Physiology, the Sahlgrenska Academy at the University of Gothenburg, Blå Stråket 15, S-413 45 Gothenburg, Sweden; 11 Core Technology Unit for Mass Spectrometry, Faculty of Medicine, Health and Life Sciences, Queen’s University Belfast, Belfast, UK

## Abstract

Delirium is a marker of brain vulnerability, associated with increasing age, pre-existing cognitive impairment and, recently, cerebrospinal fluid (CSF) biomarkers of Alzheimer’s disease. This nested case-control study used a targeted quantitative metabolomic methodology to profile the preoperative CSF of patients (n = 54) who developed delirium following arthroplasty (n = 28) and those who did not (n = 26). The aim was to identify novel preoperative markers of delirium, and to assess potential correlations with clinical data. Participants without a diagnosis of dementia (≥65 years) undergoing elective primary hip or knee arthroplasty were postoperatively assessed for delirium once-daily for three days. Groups were compared using multivariate, univariate and receiving operator characteristic (ROC) methods. Multivariate modelling using Orthogonal Partial Least Squares-Discriminant Analysis (OPLS-DA) of metabolomic data readily distinguished between delirium and control groups (R2 ≤ 0.56; Q2 ≤ 0.10). Three metabolites (spermidine, putrescine and glutamine) significantly differed between groups (P < 0.05; FDR < 0.07), and performed well as CSF biomarkers (ROC > 0.75). The biomarker performance of the two polyamines (spermidine/putrescine) was enhanced by ratio with CSF Aβ42 (ROC > 0.8), and spermidine significantly correlated with Aβ42 (pearson r = −0.32; P = 0.018). These findings suggest that spermidine and putrescine levels could be useful markers of postoperative delirium risk, particularly when combined with Aβ42, and this requires further investigation.

## Introduction

Delirium is an acute confusional state^1^ which is unpleasant for patients, their families and healthcare staff^2^. It is associated with significant negative outcomes including dementia, institutionalisation and death^3^. Postoperative delirium is common, developing after 17% of planned joint replacement surgeries^4^. Delirium is a marker of brain vulnerability, associated with increasing age, pre-existing cognitive impairment^5,6^ and, recently, cerebrospinal fluid (CSF) biomarkers of Alzheimer’s disease (AD)^7^. Whilst the pathophysiological basis for delirium remains unclear, it is thought to represent, at least partly, an aberrant stress response^8^.

Metabolomics is a relatively new technique which can comprehensively and simultaneously detect disturbances in the metabolome in a high-throughput manner^9,10^. As metabolomics directly measures chemical processes involving metabolites, it holds huge potential as a discovery platform for identifying novel diagnostic biomarkers and elucidating disease pathogenesis. The metabolomic analysis of brain tissue, CSF and plasma has advanced our understanding of the processes leading ultimately to dementia^11–13^.

Two previous studies have investigated the alteration of metabolites to samples from delirium and no delirium groups in a fracture population, one studying plasma samples^14^ and one utilising both blood and CSF^15^. Guo *et al*. reported multiple preoperative metabolic pathways disturbance in postoperative delirium groups including deficiency of ω3 and ω6 fatty acids, energy metabolism and oxidative stress with interactions between hypoxia and mitochondrial dysfunction, as well as disorders in the Glu–Gln cycle^14^. Watne *et al*. applied high performance liquid chromatography (HPLC) to measure amino acids and monoamine metabolites in CSF and serum and found increased levels of methionine, phenylalanine, tryptophan and tyrosine in fracture patients who went on to develop postoperative delirium^15^. Fracture patients are older and sicker at baseline^16,17^, have by definition already experienced trauma and often have preoperative delirium^18^. Elective orthopaedic populations, by contrast, permit comprehensive preoperative and premorbid cognitive assessment and CSF sampling at a clinically homogenous point, whilst still seeing a significant incidence of delirium.

The aim of the current nested case-control study was to employ a targeted quantitative metabolomic methodology to profile the CSF metabolome of patients who developed delirium following arthroplasty compared to those who did not. The aim was to test the hypothesis that CSF metabolomic profiles differ in those who developed delirium following arthroplasty from those who did not.

## Methods

### Study population

Participants aged 65 years or older admitted for primary elective hip or knee arthroplasty to a single surgical centre and planned to undergo spinal anaesthesia with intrathecal diamorphine analgesia were eligible for inclusion. Exclusion criteria included a pre-existing diagnosis of dementia or other neurodegenerative condition. Between March 2012 and October 2014, 315 participants completed the study. Average age was 74.4 years and 57% were female. American Society of Anesthesiologists (ASA) physical status classification was available for 53 of the 54 participants. Of these, one participant was ASA 1, 43 were ASA 2 and nine were ASA 3. The incidence of postoperative delirium in our cohort was 44/315 (14%).

### Study Methods

**Figure 1:**
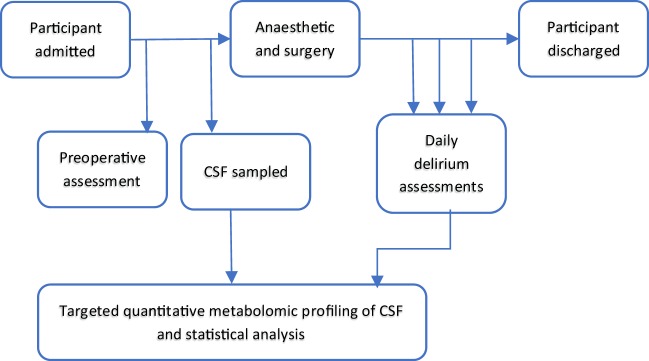
Flowchart summarising study design and methods. Matched samples n = 28 with delirium and n = 26 without delirium were used for this study.

Study methods are summarised in Fig. 1. Written informed consent was obtained from all participants and the study was approved by, and performed in accordance with, local ethical committee procedures (Office for Research Ethics Committees Northern Ireland; REC reference: 10/NIR01/5; protocol number: 09069PP-OPMS). Demographic data and potential confounding variables including age, gender, type of surgery, Charlson Comorbidity Index (CCI)^19^, level of education, estimated IQ^20^ and the Vertical Visual Analogue Pain Scores^21^ were collected preoperatively and included in analyses. Baseline neuropsychological tests included Colour Trails 2^22^ and Mini Mental State Examination^23^.

Postoperatively participants were assessed for delirium once daily by a single researcher for the first three days. Delirium was diagnosed using the Confusion Assessment Method (CAM)^24^ and daily assessments were supplemented by post-discharge reviews of the nursing and medical notes. In all cases where there were reports of possible delirium but CAM criteria were not fulfilled, cases were then discussed between ELC and APP and a consensus reached as to whether they reflected delirium according to the Diagnostic and Statistical Manual of Mental Disorders (DSM) IV criteria^25^.

### DNA collection, processing and analysis

Venous blood was sampled preoperatively into PAXgene Blood DNA tubes (PreAnalytiX; Qiagen/BD, Catalogue No. 761125). All samples were transported to the laboratory within 12 hours of collection.

DNA samples were processed according to the manufacturer’s instructions (TaqMan Single Nucleotide Polymorphism Genotyping Assay; Life Technologies; Catalogue No. 4351379).

DNA was analysed using the TaqMan Single Nucleotide Polymorphism Genotyping Assay (Life Technologies; Catalogue No. 4351379) as per the manufacturer’s instructions. *APOE* status was inferred from the genotype at each of the two alleles, rs7412 and rs429358^26^.

### CSF collection and processing

Spinal anaesthesia was carried out, fasting, in the sitting or lateral positions using 25 gauge (diameter 0.53 mm; length 90 mm) Whitacre type spinal needles with graduated metal introducers (Vygon). Once CSF was obtained, a 5 ml syringe (BD Plastipak; BD) was attached to the spinal needle and up to 5 mls of CSF withdrawn by the anaesthetist. The CSF was immediately transferred to a 30 ml sterile polypropylene universal container (Unisurge) which was placed on wet ice in an insulated container.

All samples were transported to the laboratory within 12 hours of collection. CSF samples were centrifuged at 4 °C at 3000 rpm/1811 *g* for 5 minutes with no brake and 500 mcl aliquots of the supernatant pipetted (Sarstedt, Germany, Catalogue No. 70.762) into 1.5 ml polypropylene tubes (Sarstedt, Germany, Catalogue No. 72.69.001) and stored at −80 °C.

### CSF analysis for Aβ42, T-tau and P-tau

CSF samples were transported to the Leonard Wolfson Biomarker Laboratory, University College London on dry ice. There Aβ42, T-tau and P-tau were analysed by a single, trained, technician, blinded to the clinical data, using the INNOTEST β-amyloid (1–42), hTau Ag and Phospho-tau (181 P) ELISA kits respectively (Fujirebio, Ghent, Belgium) run on a FLUOstar Omega BMG LABTECH instrument (chemiluminescent plate reader) according to the manufacturer’s protocols. Samples were thawed at 21 °C in an air-conditioned lab and vortexed prior to use. CSF biomarker values were determined using Omega software (version 5.10 R2) and a 4-parameter curve fit. Intra- and inter-assay CVs, respectively, were 2.0% and 3.9% for Aβ42, 3.3% and 8.1% for T-tau and 1.6% and 5.1% for P-tau. Longitudinal stability in the measurements is further monitored in the laboratory by participation in the Alzheimer’s Association Quality Control program^27^.

### Targeted metabolomics

Quantitative mass spectrometry-based metabolomic profiling was performed using the Biocrates AbsoluteIDQ p180 (BIOCRATES, Life Science AG, Innsbruck, Austria). All sample preparatory steps were carried out according to the manufacturer’s instructions^28,29^ and analysed on a triple-quadrupole mass spectrometer (Xevo TQ-MS, Waters Corporation, Milford, USA). The data were recorded in a 96-well format and seven calibration standards were integrated in the kit. Metabolites (amino acids and biogenic amines) were derivatised using phenylisothiocyanate (PITC) in the presence of isotopically labelled internal standards, separated using a UPLC (I-Class, Waters Corporation, UK) system with a reverse phase column (Waters ACQUITY UPLC BEH C18 2.1 × 50 mm, 1.7 μm; UK) and quantified using a triple-quadrupole mass spectrometer (Xevo TQ-MS, Waters Corporation, UK) operating in the multiple reaction monitoring (MRM) mode. All the remaining metabolites (acylcarnitines, hexoses, glycerophospholipids, and sphingolipids) were quantified using the same mass spectrometer without column separation by the flow injection analysis (FIA) operating in MRM mode. Metabolite concentrations were calculated and expressed as micromolar (µM). The mean of the coefficient of variation (CV) for the 180 metabolites in repeated quality controls was 0.12, and 85% of the metabolites had a CV of <0.15.

### Statistical analysis

#### Powering of original study

The observational cohort study was powered to detect a difference in the risk of postoperative delirium between the *APOE* ε4 positive and negative groups based on an estimated *APOE* ε4 carrier rate of 25.5%^30^ and a rate of delirium in the *APOE* ε4-positive and *APOE* ε4-negative groups of 28% and 11%, respectively, in an elective non-cardiac surgery population^31^. It was estimated that 316 participants would be required to enable detection of significant difference, at the 5% significance level, with 90% power.

#### Selection of cases and controls

Participants, with and without postoperative delirium, with sufficient CSF available to facilitate metabolomic analysis were identified. Samples for this study were selected primarily based on CSF availability. Availability of CSF was not associated with delirium in this cohort^7^. Delirium and control groups were matched for age and gender. Initially two groups of n = 27 each were identified but when additional medical notes became available and were reviewed one participant was reclassified as having had delirium using the methods outlined above, leaving n = 28 cases and n = 26 controls.

#### Included covariates

Preoperative clinical variables previously shown to be associated with delirium in our cohort (Cunningham *et al*. 2017, Cunningham *et al*. 2018) were included in the analyses. Data for perioperative intravenous opioid use was insufficient and this was therefore excluded.

#### Statistical Analysis of Metabolomic Data

Concentration data for 187 metabolites were appropriately reformatted and exported to Simca 15 (Umetrics, Umeå, Sweden) for multivariate analysis. Data were grouped into delirium and control prior to analysis by principal component analysis (PCA) identifying any potential outliers, followed by partial least squares-discriminant analysis (PLS-DA). The validity of the model was evaluated based on the residuals (R2X, R2Y) and the model predictive ability parameter (Q2) determined through the default leave-1/7th-out cross validation. The number of components for the PLS-DA model was optimized using y-table permutation testing (n = 500) and an ANOVA based on the cross-validated predictive residuals (CV-ANOVA)^32^. For CV-ANOVA assessment of significance, a p-value less than 0.05 was considered as significant. Variable importance in projection (VIP) plots was created to Identify the top metabolites responsible for discriminating between groups. The normality of data were tested using SPSS (version 24). Student’s t-test for metabolites exhibiting a normal distribution or the Wilcoxon Mann-Whitney test for metabolites exhibiting non-normal distributions, and false discovery rates (FDR, q-value) were performed to determine if metabolites were statistically different between two groups (p < 0.05, q < 0.07) (Metaboanalyst Version 3.5; (Xia *et al*., 2015). The FDR adjustment of P-values for multiple comparisons within Metaboanalyst applies the Benjamini & Hochberg correction method^33^. In order to assess and compare the biomarker performance of metabolites and/or Aβ42 receiver operating characteristic (ROC) curves were constructed (GraphPad Prism, version 6.0). This initially examined individual concentration data and subsequently assessed metabolite/Aβ42 ratios as a method of enhancing overall biomarker performance.

## Results

### Matching Of Groups

The baseline characteristics of the delirium and control groups, matched for age and gender, are shown in Table 1.

**Table 1:** Baseline characteristics for delirium and control groups.

	Delirium n = 28	Control n = 26	Statistical Test	P value
Age, mean (SD)	76.2 (5.7)	75.9 (5.2)	t = 0.224	0.82
Gender, female (%)	15/28	14/26	X2 = 0	1.00
Type of Surgery (hip vs knee), hip (%)	9 out of 28	15 out of 26	X2 = 2.604	0.11
Charlson Comorbidity Index 0/1/2/3 (md = 2)	10/12/3/1	16/8/2/0	MWU	0.09
Vertical Visual Analogue Pain Scale pain at rest, mean (SD)	19.7 (24.9)	26.5 (22.7)	t = −1.0	0.31
Apolipoprotein, ApoE4 yes (%) (md = 1)	9/27	8/26	X2 = 0	1.00
Years in Education, mean (SD)	11.3 (1.8)	11.4 (2.3)	t = −0.3	0.76
Estimated IQ, mean (SD)	106.8 (9.3)	111.7 (7.5)	t = −2.1	**0**.**04**
Mini Mental State Examination Score, mean (SD)	26.0 (2.8)	27.7 (1.9)	t = −2.5	**0**.**02**
Time taken to complete Colour Trails 2 (seconds), mean (SD)	205.2(115.1)	146.4 (50.2)	t = 2.5	**0**.**02**
Qalb, mean (SD)	6.8 (3.2)	5.9 (2.3)	t = 1.2	0.26
CSF AB42, mean (SD)	453.0(175.8)	613.9 (190.3)	t = −3.2	**<0**.**01**
CSF p-tau, mean (SD)	63.9 (23.5)	51.6 (22.0)	t = 2.0	**0**.**05**
CSF t-tau, mean (SD)	401.0(233.0)	291.3 (181.7)	t = 1.9	0.06
p-tau/AB42 ratio	0.17 (0.09)	0.10 (0.08)	t = 2.9	**<0**.**01**

### Multivariate analysis of metabolomics and clinical data

**Figure 2:**
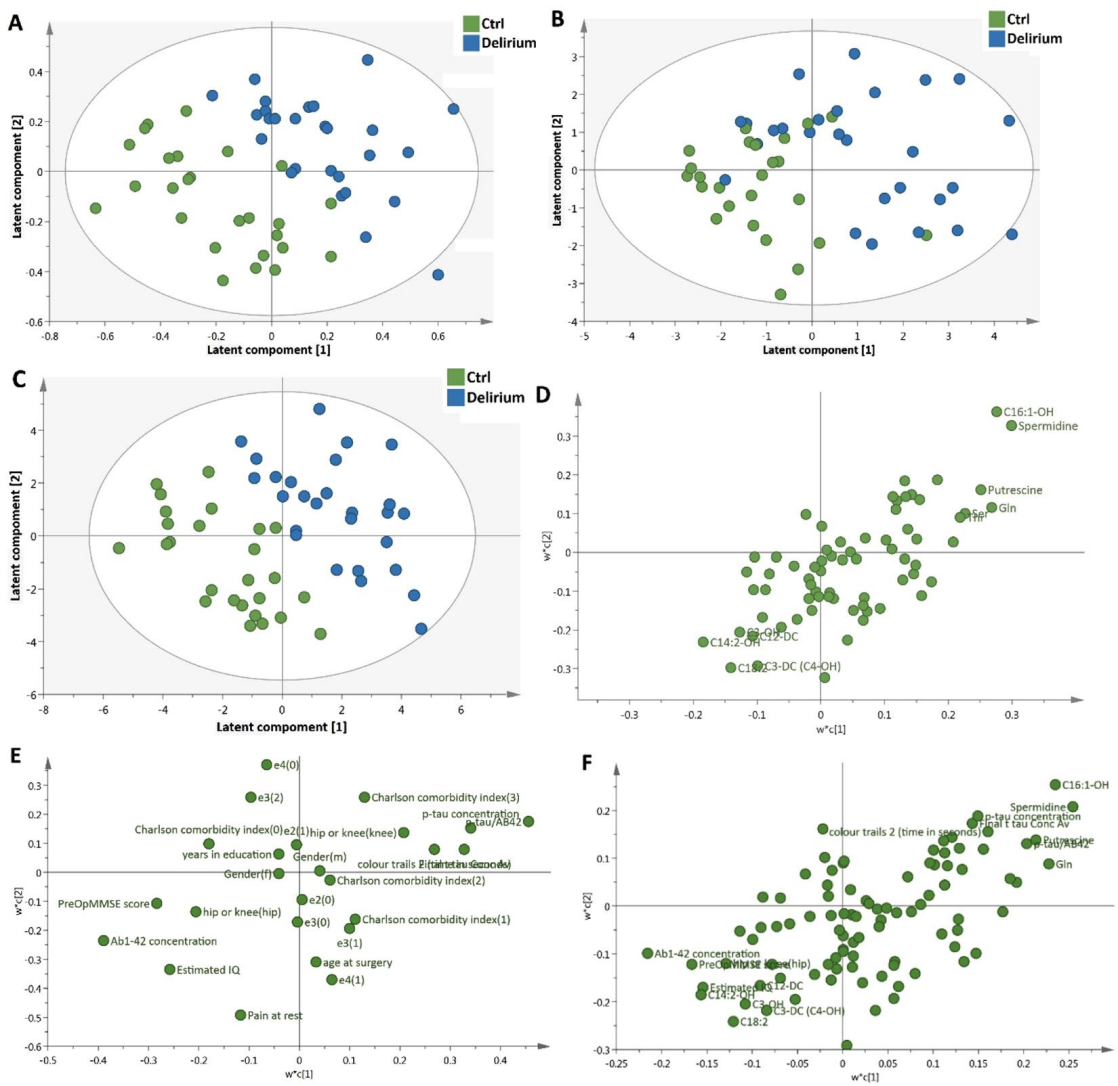
PLS-DA score plots and loadings plots derived from healthy controls and delirium patients with corresponding loadings plot. (**A** and **D**) are based on metabolomic data, (**B** and **E**) are based on pre-existing data, and (**C** and **F**) are based on combined data.

A PLS-DA model using metabolomics data only (Fig. 2A) was able to discriminate the delirium from the control subjects. With 2 components, the PLS-DA model was able to discriminate the two groups with R2X(cum) = 0.178, R2Y(cum) = 0.707, Q2 = 0.175. CV-ANOVA was used to validate the model (p = 0.016). When the same PLS-DA model was then applied to the pre-existing variables (as listed in Table 1), the PLS-DA model could only discriminate two groups with R2X(cum) = 0.266, R2Y(cum) = 0.473, Q2 = 0.123 (Fig. 2B). The p value for CV-ANOVA was 0.122 for the model, representing a statistically non-validated separation between the two groups. In addition, the predictive ability of the same PLS-DA model was significantly improved with the addition of the metabolomics data: R2X(cum) = 0.147, R2Y(cum) = 0.779, Q2 = 0.24 (Fig. 2C). The p value for CV-ANOVA was 0.79 × 10^−3^. The PLS-DA loading plots showed the most influential variables that led to the separation between two groups. Combining metabolomic and clinical data improved the predictive ability (Q2 values from 0.175 and 0.123 for metabolome and pre-existing variables respectively to 0.24 for combination) for discrimination between two groups. The model was also validated using a permutation analysis (n = 500, Supplementary Fig. 1), in which the lower Q2 intercept indicated the robustness of the model and lower risk of over-fitting. The top 20 discriminatory metabolites/pre-existing variables between two groups identified by Variable importance in projection (VIP) are shown in Table 2. The top 5 discriminatory variables consisted of 4 metabolites (spermidine, C16:1-OH, glutamine and putrescine) as well as CSF Aβ42 concentrations, of which spermidine, C16:1-OH and glutamine contributed more for discrimination between two groups than Aβ42 concentration. The top 20 discriminatory metabolites only and top 10 pre-existing variables only are shown in Supplementary Tables 1 and 2.

**Table 2:** Highest Ranking metabolites and pre-existing variables from Variable Importance Projection (VIP) based on the PLS-DA model.

	Var ID (Primary)	M3.VIP[2]	2.44693 * M3.VIP[2]cvSE
1	Spermidine	2.1055	1.50024
2	C16:1-OH	2.02904	1.87932
3	Glutamine	1.86714	1.22719
4	Ab1-42 concentration	1.76284	1.07464
5	Putrescine	1.74713	1.29829
6	p-tau/AB42	1.65976	1.16424
7	Serine	1.59627	1.19567
8	Ornithine	1.55303	0.653106
9	Alanine	1.53883	0.798499
10	Threonine	1.52848	1.15706
11	Valine	1.52002	1.14729
12	Methionine	1.47764	0.979219
13	C14:2-OH	1.38247	1.95308
14	MMSE	1.37368	1.69349
15	Colour Trails 2 (seconds)	1.35567	0.818909
16	C18:2	1.34463	1.34492
17	Estimated IQ	1.34086	1.94578
18	p-tau concentration	1.33876	1.04131
19	Lysine	1.28182	1.04599
20	C4:1	1.2796	1.95744

### Metabolites significantly altered in delirium group

**Figure 3:**
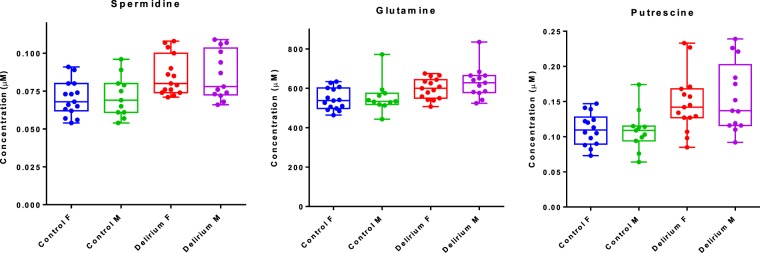
Box and whisker plots showing the three significantly altered metabolites grouped by gender.

Table 3 shows the CSF metabolites significantly altered in the delirium patients compared with control group using univariate analysis. P values from t-test, FDR q values, fold-change and ROC (AUC) were evaluated. Three metabolites (spermidine, glutamine and putrescine) were significantly different between the two groups with p values ≤ 0.05 and q values ≤ 0,07. As gender may significantly influence some metabolites^34,35^, spermidine, glutamine and putrescine are presented, in delirium and control subjects, grouped by gender in Fig. 3.

**Table 3:** List of metabolites significantly altered in the delirium group.

Metabolites	p value	FDR	↑/↓	Fold change	ROC
Spermidine	0.00014735	0.022545	↑	1.21	0.775
Glutamine	0.00090092	0.045947	↑	1.12	0.794
Putrescine	0.0017469	0.06682	↑	1.32	0.762

### Correlations between the significantly altered metabolites and Aβ42

**Figure 4:**
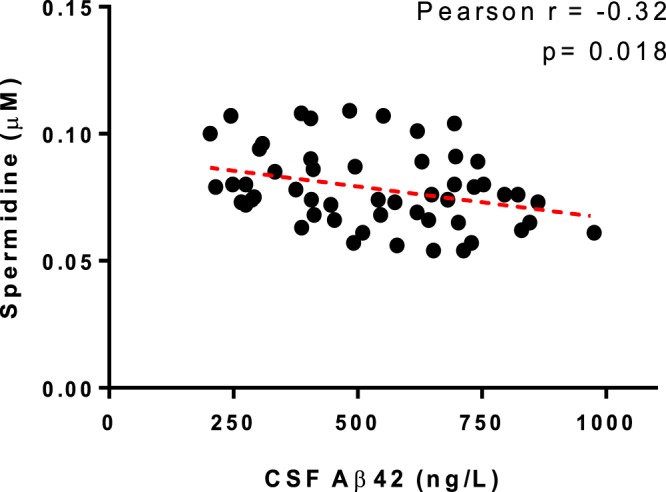
Pearson correlation analysis of the covariance between spermidine and Aβ42.

**Figure 5:**
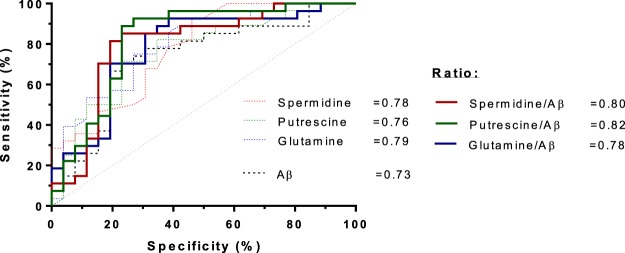
Receiving operator characteristic (ROC) curves constructed individually for all single metabolites and metabolites/Aβ42 ratio.

We reported in our previous publication^7^ that patients who developed delirium had a 26% lower CSF Aβ42 concentration in their pre-operative CSF. This was also highlighted in the VIP plots from the PLS-DA model (Table 2). Thus we evaluated the correlation between the Aβ42 and the significantly altered metabolites. Spermidine showed significantly negative correlation with Aβ42 with the Pearson r = −0.32 and p = 0.018 (Fig. 4). However, there was no significant correlation in glutamine and putrescine with Aβ42 (data not shown). The use of ratios of pairs of metabolites is a simple but powerful tool for enhancing biomarker performance^36^. Each metabolite was subjected to ratioing with Aβ42 and this led to improved AUROC values in the case of spermidine/Aβ42 and putrescine/Aβ42, but not glutamine/Aβ42 (Fig. 5).

## Discussion

We have found that three amine/polyamine compounds – spermidine, glutamine and putrescine – are higher in preoperative CSF samples from persons who went on to develop delirium postoperatively than those who did not. The addition of metabolomic data to our statistical model increased the ability of the model to identify delirium susceptible patients within a population without dementia.

The elevation of polyamine pathway metabolites has previously been reported in brain tissue, plasma and CSF from AD patients^11,37,38^. Furthermore, there is evidence that polyamines are elevated in the brain and plasma animal models developing AD pathology^12^. Interestingly, it has been shown that polyamines can interact with and promote the aggregation of Aβ peptides^39^ forming amyloid plaques, which are a hallmark of AD. Moreover, the presence of Aβ peptides appears to increase polyamine metabolism by upregulating ornithine decarboxylase activity, which leads to increased polyamine synthesis^40,41^. These results therefore complement our previous findings that lower CSF Aβ42 (reflecting accumulation of aggregated Aβ in amyloid plaques in the brain^42^) predicts postoperative delirium. Amyloid pathology causes impaired synaptic function and ultimately damages neurons, resulting in cognitive impairment and finally dementia^43^; and likely therefore increases vulnerability to the effects of stressors, including opiates, pain, and inflammatory changes seen in surgery^44^, which may result in delirium.

Understanding the biochemical basis for the development of postoperative delirium is a necessary first step if we are to intervene to reduce the incidence of this common, unpleasant and potentially dangerous syndrome. Identifying the pathways responsible may also enhance our understanding of neurodegeneration which predisposes to delirium. The study of elective orthopaedic populations permits comprehensive preoperative and premorbid cognitive assessment in contrast to research undertaken in fracture populations when participants are older and sicker at baseline^16,17^, have by definition already experienced trauma and often have preoperative delirium^18^. The incidence of delirium is sufficiently high to study this condition^4^, but low enough to likely indicate an underlying vulnerability. Although the findings of this nested case-control study are based on a relatively small sample and are not immediately translational, the key strengths include the use of fasting samples (all samples were preoperative), the detailed clinical information, and the annotated and quantitative metabolomic data.

## Conclusions

This study indicates the benefits of applying metabolomic methodologies to conditions such as delirium to determine susceptibility risk: our findings suggest that CSF levels of spermidine, putrescine, and their metabolite precursor glutamine, could be useful markers of postoperative delirium risk, particularly when combined with Aβ42. The development of rapid and inexpensive methods of measuring spermidine and putrescine is needed to evaluate their utility in larger prospective patient cohorts, and to study whether CSF amines/polyamines are markers of underlying neurodegeneration (i.e. preclinical Alzheimer’s disease), or for delirium *per se*. Replication of these findings, in both delirium and AD cohorts, is now necessary.

## Supplementary information

Cerebrospinal Fluid Spermidine, Glutamine and Putrescine Predict Postoperative Delirium Following Elective Orthopaedic Surgery

## Data Availability

The raw metabolomic data will be provided with this manuscript on request. After publication it will be submitted to an online data repository such as Metabolights. Readers can obtain further information on correspondence with the authors.
