# Supplementary material for: Cerebrospinal Fluid Spermidine, Glutamine and Putrescine Predict Postoperative Delirium Following Elective Orthopaedic Surgery
Source: Sci Rep. 2019 Mar 12;9:4191. doi: 10.1038/s41598-019-40544-3 (PMC6414730; doi:10.1038/s41598-019-40544-3)
Supplement: Supplementary file 1 — Cerebrospinal Fluid Spermidine, Glutamine and Putrescine Predict Postoperative Delirium Following Elective Orthopaedic Surgery [file 41598_2019_40544_MOESM1_ESM.pdf]

Supplementary Information

Cerebrospinal Fluid Spermidine, Glutamine and Putrescine Predict Postoperative Delirium Following Elective Orthopaedic Surgery

Authors

*Joint first author

Xiaobei Pan, PhD*

Emma L Cunningham, PhD MRCP*

Anthony P Passmore, MD FRCP

Bernadette McGuinness, MD PhD FRCP

Daniel F McAuley, MD FRCP

David Beverland, MD FRCS

Seamus O'Brien, PhD

Tim Mawhinney, BSc

Jonathan M Schott, MD FRCP

Henrik Zetterberg, MD PhD

Brian D Green, PhD

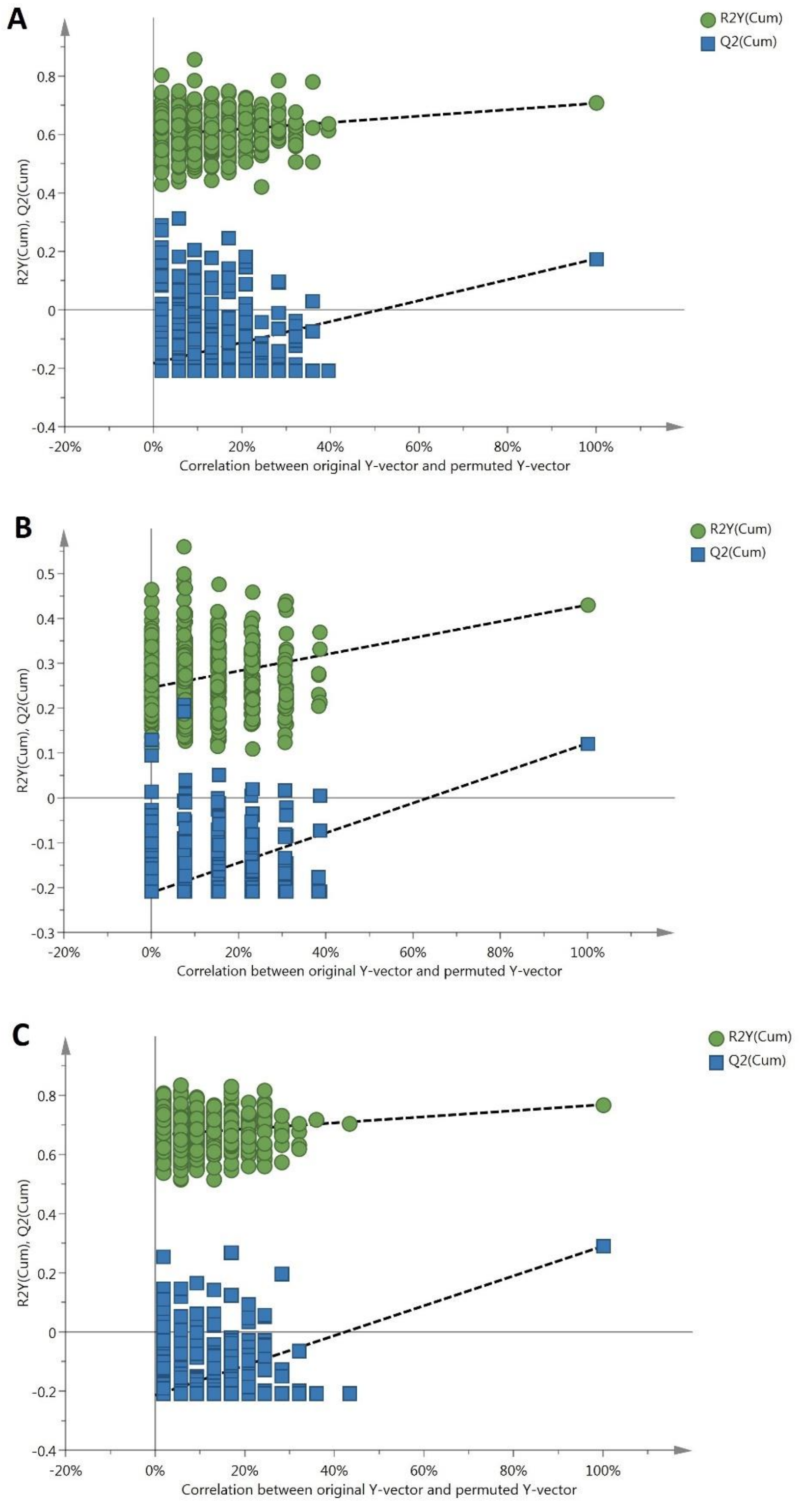

Supplementary Figure 1 Permutation plots (n=500) for validating the PLS-DA model building with (**A**) metabolomics data only; (**B**) clinical variables only and (**C**) both metabolomics and clinical variables.

Supplementary Table 1 Highest Ranking metabolites from Variable Importance Projection (VIP) based on the PLS-DA model

	Var ID (Primary)	M4.VIP[2]	2.44693 * M4.VIP[2]cvSE
1	C18:1-OH	2.2773	1.7268
2	C16:1-OH	1.87861	1.14876
3	C5-M-DC	1.83137	1.49098
4	Putrescine	1.81183	2.2533
5	Spermidine	1.74598	0.926234
6	C4:1	1.57793	2.98705
7	C9	1.56977	1.52801
8	C3-OH	1.53123	0.982301
9	C3-DC (C4-OH)	1.43514	1.32952
10	C5:1-DC	1.39364	1.62419
11	C18:2	1.39044	1.21586
12	C14:2	1.3651	2.12922
13	C10:2	1.36082	0.983162
14	Thr	1.29975	0.282446
15	Orn	1.28524	1.03495
16	C5-OH (C3-DC-M)	1.19356	1.0874
17	Gln	1.19055	0.792551
18	Ala	1.16119	2.05692
19	Val	1.08643	0.691744
20	Ser	1.06461	0.938487

Supplementary Table 2 Highest Ranking clinical variables from Variable Importance Projection (VIP) based on the PLS-DA model

	**Var ID (Primary)**	**M2.VIP[2]**	**2.44693 * M2.VIP[2]cvSE**
1	p-tau/AB42	1.99312	0.93908
2	Ab1-42 concentration	1.70869	1.01552
3	p-tau concentration	1.48805	1.31182
4	t-tau concentration	1.43419	1.19531
5	Estimated IQ	1.26214	1.09553
6	MMSE	1.24328	1.01909
7	Pain at rest	1.20078	1.92492
8	Colour Trails 2 (seconds)	1.17676	1.28322
9	e4(0)	1.00127	0.430106
10	e4(1)	1.00127	0.430106
